# Current Status of Childhood Hyperinsulinemic Hypoglycemia in Turkey

**DOI:** 10.4274/jcrpe.2991

**Published:** 2016-12-01

**Authors:** Zeynep Şıklar, Merih Berberoğlu

**Affiliations:** 1 Ankara University Faculty of Medicine, Department of Pediatric Endocrinology, Ankara, Turkey

**Keywords:** Hyperinsulinism, hypoglycemia in infancy, congenital hyperinsulinism, hyperinsulinemic hypoglycemia

## Abstract

Congenital hyperinsulinism (CHI) is a rare disease characterized by dysregulated insulin secretion from pancreatic β-cells. Recurrent hypoglycemia can lead to neurological insult and permanent brain injury. Recently, there are important advances in understanding the genetic mechanisms, histological characteristics, imaging, and surgical techniques of congenital hyperinsulinemic hypoglycemia that could reflect to improvement in the clinical care of infants with this disorder. In Turkey, there is a high rate of consanguinity, thus, the incidence of CHI is expected to be high. Until now, there are no nationwide data regarding the disorder, and some individual case reports or case series had been published. Determining the characteristics of Turkish patients with CHI can help develop a different perspective on this rare disease. In this review, we evaluated the clinical and molecular characteristics of Turkish patients with CHI based on reports published in the literature. The most frequently seen mutations were ABCC8 gene mutations (n=37), followed by HADH (n=11) and KCNJ11 gene (n=7) mutations. A total of 141 Turkish patients with CHI were reported until now. Among them, 115 patients had been genetically analyzed, and 56 of them had one of the mutation leading to hyperinsulinism.

## INTRODUCTION

Hyperinsulinemic hypoglycemia (HH) consist of a group of heterogeneous disorders characterized by unregulated insulin secretion from pancreatic β-cells (1). It is the commonest cause of both persistent and transient hypoglycemia in neonates and infants (2,3,4). Because there can be severe brain damage related to hypoglycemia, it is vital to diagnose and treat patients with HH correctly (1,2,3,4).

The clinical presentation of HH can be very heterogeneous ranging from very subtle presentation to severe disease needing pancreatectomy (2,3,4,5). It can be either permanent or transient; the permanent form is usually known as congenital. Transient forms of congenital hyperinsulinism (CHI) usually occur in newborns with certain risk factors like maternal diabetes mellitus, intrauterine growth retardation, perinatal asphyxia. Some case with transient HH might have HNF4A gene, HNF1A gene, or ATP-sensitive potassium (K_ATP_) channel mutations.

CHI is the most severe and persistent form of hereditary HH (1,2,3,4). Persistent CHI can be caused by mutations in nine genes regulating the insulin secretion from the β-cells (ABCC8, KCNJ11, GLUD1, HADH, GCK, HNF4A, HNF1A, SLC16A1, and UCP2 genes) (6,7). The most common causes are mutations in the ABCC8 and KCNJ11 which encode the SUR1 and Kir6.2 subunits of the pancreatic β-cell K_ATP_ channel (2,3,4). K_ATP_ channels in pancreatic β-cells regulate the flux of K ions across cell membranes. Glucose phosphorylation by glucokinase controls glucose-regulated insulin secretion. Glucose 6 phosphatase produces ATP by getting metabolized. This leads to an increase in intracytosolic ATP:ADP ratio. Increased ATP:ADP ratio inhibits the activity of the K_ATP_ channel. Following from this, ATP channel gets closed and membrane depolarization occurs. From this point on, voltage-dependent calcium channels open resulting in more calcium going into the beta cells. Higher concentrations of calcium trigger secretory granules to release insulin (8,9).

Mutations in ABCC8 and KCNJ11 can be either autosomal-recessive or autosomal-dominant (10).

Histologically, CHI is classified in three subgroups: diffuse, focal, and atypical forms. Most of the times, diffuse form is inherited autosomal-recessively. Autosomal dominant hereditation is also seen but not as often. Focal form, which occurs sporadically, can be seen with the combination of paternal heterozygous germline mutation in one of the ABCC8 and KCNJ11 genes and uniparental disomy of the maternal chromosome 11p15. In patients with atypical disease, the histological abnormalities may be diffuse with coexistence of normal and abnormal islets (9).

The incidence of CHI was reported as 1 in 40 000 in the general population to 1 in 2500 in certain communities (11). In Turkey, there is a high rate of consanguinity, thus the incidence of CHI is expected to be high (12,13). Until now, there are no nationwide data regarding this disorder, and only some individual case reports and studies including a total of 141 patients had been published (6,13,14,15,16,17,18, 19,20,21,22,23,24,25,26,27,28,29,30,31,32). Determining the characteristics of Turkish patients with CHI may gain a different perspective to this rare disorder.

In this review, we aimed to evaluate the all published papers reporting Turkish patients with CHI and to explore both diagnostic and follow-up characteristics of these patients.

## METHODS

The PubMed, SCOPUS, and Web of Science electronic databases were systematically searched from inception to February 10, 2016. The search terms were “hyperinsulinism” or “CHI” or “nesidioblastosis” or “hypoglycemia” or “persistent hyperinsulinemic hypoglycemia of infancy” or “hyperinsulinemic hypoglycemia of infancy” or “HI” or “hyperinsulinaemic” or “hyperinsulinemic” and “Turkey” or “Turkish”. No search filters or language restrictions were imposed. All case reports, case series, and studies on Turkish patients with HH were evaluated. Papers reporting other than congenital or persistent HH were excluded from the analysis. The data of cases in authors’ center were also given.

## GENERAL CHARACTERISTICS OF PATIENTS

A total of 18 manuscripts reporting Turkish patients with CHI had been published. A total number of 12 case reports including 1 to 3 patients were published between 1997 and 2016. Because some patients were presented in different papers, we counted them once. Only six papers reporting 4 to 35 patients’ data had been published, including some patients individually reported before. The number of all reported Turkish patients was 141. Among them, 115 patients had undergone molecular genetic analysis, and 56 of them had one of the mutations leading to hyperinsulinism. A total of 26 patients had not undergone molecular studies (6,13,14,15,16,17,18,19,20,21,22,23,24,25,26,27,28,29,30,31,32).

## ADMISSION CHARACTERISTICS OF TURKISH PATIENTS

It was reported that the most common presentation of HH is during the neonatal period (1,2,3,4). These finding is similar to the characteristics of Turkish patients. The majority of reported Turkish patients were diagnosed during the neonatal period.

Delivery of macrosomic fetus is a sign of intrauterine hyperinsulinemic state. Among the reported Turkish cases, the rate of macrosomic babies was 34%.

## ETIOLOGY OF TURKISH HYPERINSULINEMIC HYPOGLYCEMIA OF PATIENTS

HH can be transient. Among the causes for transient form of HH, maternal diabetes mellitus, intra-uterine growth retardation, perinatal asphyxia, erythroblastosis fetalis, maternal administration of drugs, intravenous glucose infusions during delivery, as well as Sotos syndrome can be counted (10,33). There were only two papers reporting prolonged transient HH in Turkish children (15,32). Although transient HH usually resolves spontaneously in a few weeks, in some patients, the duration of hyperinsulinism can be protracted, requiring diazoxide treatment (10). In those patients, the mechanism for hyperinsulinism is not clear. Güven et al (32) reported a case series of CHI patients, and in 7 of 13 patients which responded to diazoxide therapy, treatment had been stopped between 15 days to 12 years. Ağladıoğlu et al (15) presented 17 HH cases in their series, eight of which had transient HH; diazoxide therapy had been ceased between 3 to 154 months. No etiological or molecular genetic studies were done in these patients.

Recently, it has appeared that some transient HH patients have HNF4A gene, HNF1A gene, or K_ATP_ channel mutations (16,34). HNF4-MODY and HNF1A-MODY were well-known subtypes of autosomal-dominant diabetes. Early postnatal HH could be accompanied in individuals with those mutations (34).

Huopio et al (35) described the first dominantly inherited ABCC8 mutation (E1507K, previously reported as E1506K) that caused HH in early life and predisposed to early-onset insulin deficiency. It was suggested that some adults with a dominant K_ATP_ channel mutation were asymptomatic, and these mutations could have been missed in infancy (36).

We followed up two siblings with transient HH who were heterozygous for ABCC8 missense mutation, A1367D (c.4100C>A; p.Ala1367Asp). The clinical presentation of these patients with a dominant ABCC8 mutation was milder than that of patients with the recessive form of the disease; they responded well to medical therapy. Both siblings have been diagnosed with autoantibody-negative diabetes mellitus during the prepubertal period following a remission of the HH in childhood. Their mother, maternal aunt, and maternal grandfather were also heterozygous for the same mutation. The mother was diagnosed with type 2 diabetes mellitus at the age of 28 years, and the maternal aunt and grandfather had a medical history of postprandial hypoglycemic attacks; there was no family history of hypoglycemia in neonatal period.

Another interesting patient that we diagnosed as transient HH was born after caesarian delivery with birth weight of 4250 g. He had been diagnosed with nonketotic HH at 2 days of life (when blood sugar was 38 mg/dL and serum insulin level was found as 7.3 mIU/mL). The patient responded well to diazoxide treatment in a dose of 10 mg/kg/day. During follow-up, diazoxide dose was tapered gradually and ceased at 3 months of life. Now, he is 2.5 years of age and good in condition. He was heterozygous for a novel KCNJ11 gene missense mutations and a KCNJ11 frameshift mutation [Exon1B/Exon1C;c.130G>A/c.405dup; p.Val144Met/p.Arg136fs (p.V44M/p.R136fs)]. The patient had inherited the p.V44M mutation from his clinically unaffected mother.

In the light of these findings, it should be kept in mind that transient HH in infancy can be related to K_ATP_ channel mutations, and diabetes mellitus may develop later in life.

## GENETIC MUTATIONS OF PATIENTS

**Figure 1:**
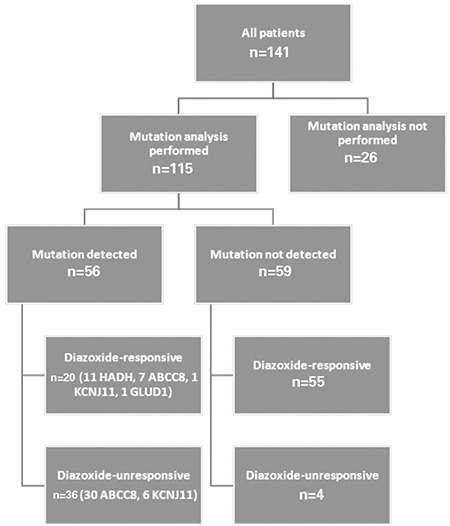
Distribution of patients according to mutation analysis results and diazoxide responsiveness

Molecular studies showed that the congenital form of HH is due to mutations in eight different genes (ABCC8, KCNJ11, GLUD1, CGK, HADH, SLC16A1, HNF4A, and UCP2). Most of the CHI cases are caused by K_ATP_ channel mutations. K_ATP_ channels in pancreatic β-cells are composed of four inward-rectifying potassium channel (Kir6.2) subunits and four high-affinity sulfonylurea receptor 1 (SUR 1) subunits (37). After excluding the recurrent reported cases, a total of 115 cases had been analyzed for genetic mutations; in 26 cases, no genetic analysis had been carried out. 44 of patients with mutation analysis done had K_ATP_ channel genes mutations (Figure 1). The most frequently seen mutations were ABCC8 gene mutations (n=37), followed by HADH (n=11), KCNJ11 (n=7) and GLUD1 (n=1) gene mutations (6,13,14,15,16,17,18,19,20,21,22,23,24,25,26,27,28,29,30,31,32).

ABCC8 gene mutations have been reported as the commonest cause of CHI by Demirbilek et al (24) in the largest cohort consisting of Turkish CHI patients. They estimated the frequency of ABCC8 mutation in the CHI patients as 40% (14/35). In this cohort, eight different ABCC8 mutations had been identified. One of the commonest mutations in their cohort (5/14) was p.L1171fs (c.3512del), a frameshift mutation on exon 28 of ABCC8 gene. Güven et al (32) also reported that 9 of 12 mutation-positive patients had ABCC8 gene mutation.

KCNJ11 gene mutations had been detected in seven patients (7/56). Three of them were reported in a cohort (24). The authors of this review detected three patients with KCNJ11 mutation. All except one patient with this mutation were diazoxide-unresponsive and required pancreatectomy. One diazoxide-unresponsive patient with KCNJ11 gene mutation responded well to octreotide therapy (32).

A female patient’s follow-up in the authors’ center revealed two novel mutations in the KCNJ11 gene in exon 1 which were paternally inherited (p.R221H and p.Q299H). Her father was not clinically affected and mother had no K_ATP_ channel mutation. Interestingly, in this case, maternal loss of heterozygosity between chromosomes 11p15.5 and 11p15.1 covering the region (mosaic uniparental disomy) that may lead to Beckwith-Wiedemann syndrome was found. In histopathological evaluation, diffuse lesions were detected.

The distinction between focal and diffuse forms of hyperinsulinism cannot be made by clinical or biochemical means in cases with CHI. Positron emission tomography/computed tomography by 18 fluoro-L-DOPA (^18^F-DOPA PET/CT) is a noninvasive scanning tool with the capability to distinct between focal and diffuse forms (38). Unfortunately, ^18^F-DOPA PET/CT scanning is not available in Turkey.

We observed that HADH gene mutations were relatively frequent in Turkish CHI patients, accounting for 20% (11/56) of all mutation-studied cases (6,13,24,32), despite the fact that the mutations in that gene are reported as a rare cause of recessively inherited HH (7,10). HADH gene encodes the mitochondrial enzyme L-3-hydroxyacyl-coenzyme A dehydrogenase (HADH). Loss-of-function mutations in the HADH gene cause short-chain L-3-hydroxyacyl-CoA (SCHAD) deficiency. The mechanism behind unregulated insulin secretion in SCHAD deficiency is not fully understood but may involve changes in protein-protein interactions with glutamate dehydrogenase (GDH) (6).

These patients exhibit severe protein (especially leucine) sensitivity, with some subjects having raised plasma levels of hydroxybutyrylcarnitine as well as elevated urinary levels of medium-chain dicarboxylic and 3-hydroxydicarboxylic metabolites and 3-hydroxyglutarate (6). The clinical presentation is mainly neonatal- or early infancy-onset CHI, but mild late-onset phenotype can be seen (6,10). So far, approximately 40 patients with CHI resulting from a mutation in the HADH gene have been reported, and all of them have responded well to diazoxide therapy (6).

Because the consanguinity is high in the Turkish population, occurrence of recessively inherited HADH gene mutation in increased rate seems to be inevitable. It is convenient that sequencing of HADH gene be recommended in all patients with diazoxide-responsive HH.

The method for detection of HADH gene mutation is also important which could be different in the centers. Most of the Turkish patients (n=8) with HADH gene mutation were reported by Flanagan et al (13). They detected deep intronic mutations of that gene by using next-generation sequencing analysis which have not been demonstrated before by Sanger sequencing.

Among the reported Turkish patients, there was no case with exercise-induced hyperinsulinism (SLC16A1), glucokinase-induced hyperinsulinism, or mutations in the UCP2, HNF4A, and HNF1A genes, while one patient had GLUD1 gene mutation (32).

## TREATMENT MODALITIES OF TURKISH PATIENTS

For preventing hypoglycemia-related irreversible brain damage, aggressive and early intervention remains the mainstay of treatment in HH. The first-line drug management of persistent CHI is diazoxide therapy. Diazoxide binds to SUR1 component of K_ATP_ channels resulting in their opening. It is effective in most of the CHI forms except in those due to recessive (and some dominant) inactivating mutations in ABCC8 and KCNJ11 and in patients with focal CHI (10). The second-line medical treatment is octreotide therapy. If octreotide fails to control the hypoglycemia, surgical intervention is usually required.

Most of the Turkish patients with CHI were responsive to diazoxide treatment (100/141, 71%), while 18 of them had the transient form. Among patients with reported genetic mutation analysis (n=115), 75 were diazoxide-responsive.

All Turkish patients with HADH gene mutation were diazoxide-responsive, thus no surgical treatment was applied, as expected. Pancreatectomy was implemented in 28 of diazoxide-unresponsive CHI patients which accounts for 19.8% of all CHI patients. All of them, except one, had K_ATP_ channel mutations.

Pathological examination of pancreatic tissues revealed the diffuse form of hyperinsulinism in 27 of 28 cases. Moreover, the focal form of hyperinsulinism has been reported in the literature in almost half of all patients treated surgically (7); only one patient with focal form has been reported from our country (32).

Because ^18^F-DOPA PET/CT is not available in our country, genetic analysis would be important to discriminate the focal disease before the decision of pancreatectomy. Even in patients with mutation analysis indicating focal disease, this form might not be pathologically proven in every case. It is suspected that: Focal disease might not really have been absent among the patients. The pathological examination might have been insufficient in some patients.

It has been reported that pancreatectomy is associated with a high incidence of diabetes mellitus and pancreatic exocrine insufficiency. For that reason, surgical treatment should be reserved for the cases with unsuccessful medical treatment (37,39). Among the reported Turkish patients with CHI, there is very scarce long-term information after pancreatectomy with aspect to development of diabetes and other possible complications. In a case series, follow-up time was relatively low as near 6 years (32). In addition, there is no knowledge about disease-free survival. It would be important to demonstrate the follow-up of characteristics revealing the improvement in the management of these patients.

Very recently, a new medical treatment option has emerged for diffuse CHI unresponsive to diazoxide and/or octreotide treatment. The mammalian target of rapamycin inhibitor sirolimus has been successfully used in some diazoxide- and octreotide-unresponsive CHI patients. It reduces the pancreatic B-cell proliferation and inhibits insulin production (40).

Neurological outcome: One of the most important long-term complications of CHI is severe brain damage such as cerebral palsy, epilepsy, developmental delay (1,2,3). Among the reported cases from Turkey, there are no extensive data about long-term neurological consequences. From the two largest series, it was seen that neurological sequelae were encountered in almost one third of patients (34% and 29%) (15,24). Especially diazoxide-unresponsive patients were under high risk for development of neurological sequelae.

The most favorable neurological outcomes were seen in patients with HADH gene mutation. The longest follow-up was reported in a female patient with HADH gene mutation who had no neurological findings (6).

Early and aggressive treatment of patients with severe CHI is necessary to prevent brain damage, and diazoxide responsiveness gives an important clue for good prognosis and further treatment.

In conclusion, CHI is a heterogeneous disorder. K_ATP_ channel mutation is the most frequent etiological factor among mutation-studied Turkish patients with CHI. Patients with HADH gene mutation are relatively frequent among them. Transient form of HH in infancy could be caused by K_ATP_ channel mutations, and diabetes mellitus may develop later in life. Because long-term neurological damage is high in diazoxide-unresponsive patients, early and prompt intervention is needed in such patients. There is no clear information and follow-up data, including development of diabetes, about patients who have undergone pancreatectomy. Multicenter studies are needed to obtain long-term follow-up characteristics of such patients at national base.

## Ethics

Peer-review: Externally peer-reviewed.
